# A systematic review of demographic and background factors associated with the development of children’s aquatic competence

**DOI:** 10.1186/s40621-023-00447-4

**Published:** 2023-08-08

**Authors:** Charlotte Duke, Hannah Calverley, Lauren Petrass, Jacqui Peters, Kate Moncrieff, Loretta Konjarski, Bernadette Matthews

**Affiliations:** 1 Life Saving Victoria, Melbourne, Australia; 2 Institute of Education, Arts and Community, Federation University, Ballarat, Australia; 3 Deakin University, Melbourne, Australia; 4 Victoria University, Melbourne, Australia; 5 School of Public Health and Preventive Medicine, Monash University, Melbourne, Australia

**Keywords:** Aquatic competency, Swimming skill, Social determinants, Drowning prevention, Swimming and water safety, Aquatic education

## Abstract

**Background:**

Globally, drowning is a leading cause of unintentional injury and death among children. Teaching aquatic competencies (swimming skills and water safety knowledge) to children has been proposed as a prevention strategy. In Australia, however, many children are not meeting standard aquatic competency benchmarks. Exploration of the connection between demographic and background factors and aquatic competencies could provide insight into why differences in acquisition of aquatic knowledge and skills occur.

**Main body:**

A systematic literature review guided by the Preferred Reporting Items for Systematic Reviews and Meta-Analyses was performed to identify studies that reported on the association between demographic and background factors and aquatic competencies. Nine databases were searched for English language peer-reviewed studies published since 2000. Fourteen studies fulfilled all inclusion criteria. Studies were quasi-experimental or cross-sectional in design, which is considered quality level III-2 or IV, respectively, on the National Health and Medical Research Council Evidence Hierarchy. Study quality was moderate, and risk of bias was high. While aquatic competencies can be taught, this review found that factors including age, gender, geographic residence, medical conditions/disabilities, socioeconomic status, and swimming frequency were significantly associated with the demonstration and/or acquisition of aquatic competencies.

**Conclusion:**

This review provides insight into demographic and background factors that are significantly associated with the development of aquatic competence. Whilst further investigation is required to increase the evidence base, these findings may assist in tailoring swimming and water safety programs to accommodate those at-risk of not achieving age-appropriate aquatic competencies.

**Supplementary Information:**

The online version contains supplementary material available at 10.1186/s40621-023-00447-4.

## Background

Drowning is one of the leading causes of death globally and disproportionally impacts young children aged 1–14 years (World Health Organisation 2017). Improving children’s aquatic competence (the ability to perform specific swimming and water safety skills) is an accepted child drowning prevention strategy (Taylor et al. 2020). Accordingly, swimming and water safety education programs are strongly recommended for children, both by the World Health Organization (WHO) (2017) and the Australian Water Safety Council (2021). However, Stallman et al. (2017) argued that only physical competencies (such as swimming ability) are insufficient to protect against drowning in isolation. When performed alongside cognitive and affective competencies (such as water safety knowledge, behaviours and attitudes), a person attains aquatic competence. Empirical evidence on the direct connection between aquatic competence and drowning risk remains inconclusive (Brenner et al. 2003). Some studies examining attitudes of older adolescents and adults found that compared to weak swimmers, stronger swimmers were more likely to take risks around water or visit dangerous aquatic environments, which increases drowning risk (Brenner et al. 2003; McCool et al. 2008; Brenner et al. 2006). Conversely, other studies have reported that swimming ability and water safety knowledge may protect against drowning (Brenner et al. 2009; McCool et al. 2009). A recent systematic review identified that young children (aged 2–4 years) who participated in formal swimming lessons were more likely to be adequately supervised and the acquisition of aquatic competencies was of benefit for the prevention of drowning (Taylor et al. 2020). The authors acknowledged, however, that included studies were limited by small sample sizes and heterogeneous definitions of aquatic competence.

In Australia, school-based lessons provide one avenue for children attending primary school, aged 5–12 years, to develop aquatic competence. Such lessons are usually aligned to the Health and Physical Education learning area of the Australian Curriculum Assessment and Reporting Authority (2022) and the Royal Life Saving Australia (2019). These standards include competencies for swimming, lifesaving, rescue, and survival skills, as well as water safety knowledge. The duration, structure, content, and availability of school-based lessons differ between schools and across the country (Petrass et al. 2021). One reason for this is that aquatic programs are designed to meet the learning needs and strengths of individuals and/or groups of students. Differentiated instruction can involve adaptations to the lesson content (what students learn), the process (how students learn), or the product (the learning environment) (Gibbs and McKay 2021; Tomlinson 2014). Differentiated aquatic instruction is particularly evident for children with disabilities (Pearn and Franklin 2013), and for children of migrant and culturally and linguistically diverse backgrounds (Savage and Franklin 2015; Lõhmus et al. 2022). However, even experienced teachers have found differentiating to meet the needs of all the students in swimming classes challenging (Whipp et al. 2014). Therefore, in addition to lessons provided through schools, parents of some children also pay for private swimming lessons with the commercial swim industry to develop swimming and water safety skills and knowledge (Willcox-Pidgeon et al. 2021).

The commercial swim industry has experienced exponential growth in the last decade (Peden and Franklin 2012). However, there is limited published evidence reporting the level of aquatic competency children develop through these programs. A retrospective cross-sectional study explored 42,201 records (extracted from the National Swimming and Water Safety Framework database) from Australian children aged 5–12 years attending commercial swim lessons across New South Wales, Victoria, and South Australia (Willcox-Pidgeon et al. 2021). Results indicated that among 12-year-old children, 60.3% of the cohort achieved the 50 m freestyle benchmark, and a third achieved the benchmark for the survival skill ‘sculling’ or treading water for a minimum of two minutes. Children of low socioeconomic status (SES) and those living in a regional area (towns, small cities and areas that lie beyond major capital cities) were significantly less likely to be achieving the swimming and water safety benchmarks (Willcox-Pidgeon et al. 2021). These findings highlight inequalities with respect to achievement levels and ease of accessing commercial swimming lessons (Peden and Franklin 2012). Therefore, further research is required to systematically investigate the association of demographic and background factors with the acquisition of aquatic competencies to ensure children of lower aquatic competence are supported in achieving essential swimming skills (Willcox-Pidgeon et al. 2021; Pharr et al. 2018; Peden et al. 2017). Accordingly, the aims of this systematic review were to:

1. Identify the demographic and background factors associated with children’s aquatic competence in high-income countries.
2. Assess the quality of evidence available regarding demographic and background factors and children's aquatic competence.
3. Determine the demographic and background factors that should be considered when addressing children’s aquatic competence.

## Main text

### Methods

This systematic review followed the Preferred Reporting Items for Systematic Reviews and Meta-Analyses (PRISMA) guidelines (Page et al. 2021).

### Data sources and search strategy

We systematically searched peer-reviewed literature, published from 2000 until January 2022 (inclusive), for work that identified relationships between demographic and background factors and aquatic competence in children aged 12 years and under. For this research, aquatic competence encompassed both water safety knowledge and swimming ability, as defined by the Victorian Water Safety Certificate (VWSC; the Victorian standard for swimming and water safety, which aligns with the national standards) (Life Saving Victoria 2022).

Eight databases (Medline, Embase, PsycINFO, CINAHL complete, PubMed, Scopus, ProQuest, A + Education) and Google Scholar were searched. These were selected as they provided disciplinary breadth covering injury prevention, drowning prevention, and education. In addition, a snowballing method searching the bibliographies of retrieved references was applied to identify further potentially relevant articles. Only English search terms were used, with wildcards, related terms, and truncation search features implemented. We divided search terms into three groups, with terms within groups combined using the Boolean operator OR, and groups combined using the Boolean operator AND.

1. Drown* OR drowning prevention OR drowning intervention OR water safety AND
2. Swim* ability OR aquatic development OR aquatic achievement OR (aquatic AND competenc*) OR (aquatic AND literacy) OR swim* lessons AND
3. Child* OR adolescent* OR youth* or teenager*

### Study selection: inclusion and exclusion criteria

The inclusion and exclusion criteria were developed a priori. Studies were eligible for inclusion if they were: (i) peer-reviewed and available as full text; (ii) published in English or an English translation was available; (iii) conducted in a high-income country (HIC); (iv) provided a direct measure of aquatic competence, not including self-reported or parent-reported competence; (v) reported on participants aged 12 years and younger; and (vi) presented the relationship between aquatic competence and demographic and background factors. If the average age of children reported in a study was under the age of 12 years, the study was included. Studies were excluded if they: explored drowning risk, not aquatic competence; explored outcomes only in adolescents older than 12 years; and reported findings from low- and middle-income countries (LMIC) where there is large variability in aquatic education provision, compared to HIC (World Health Organisation 2017).

**Fig. 1:**
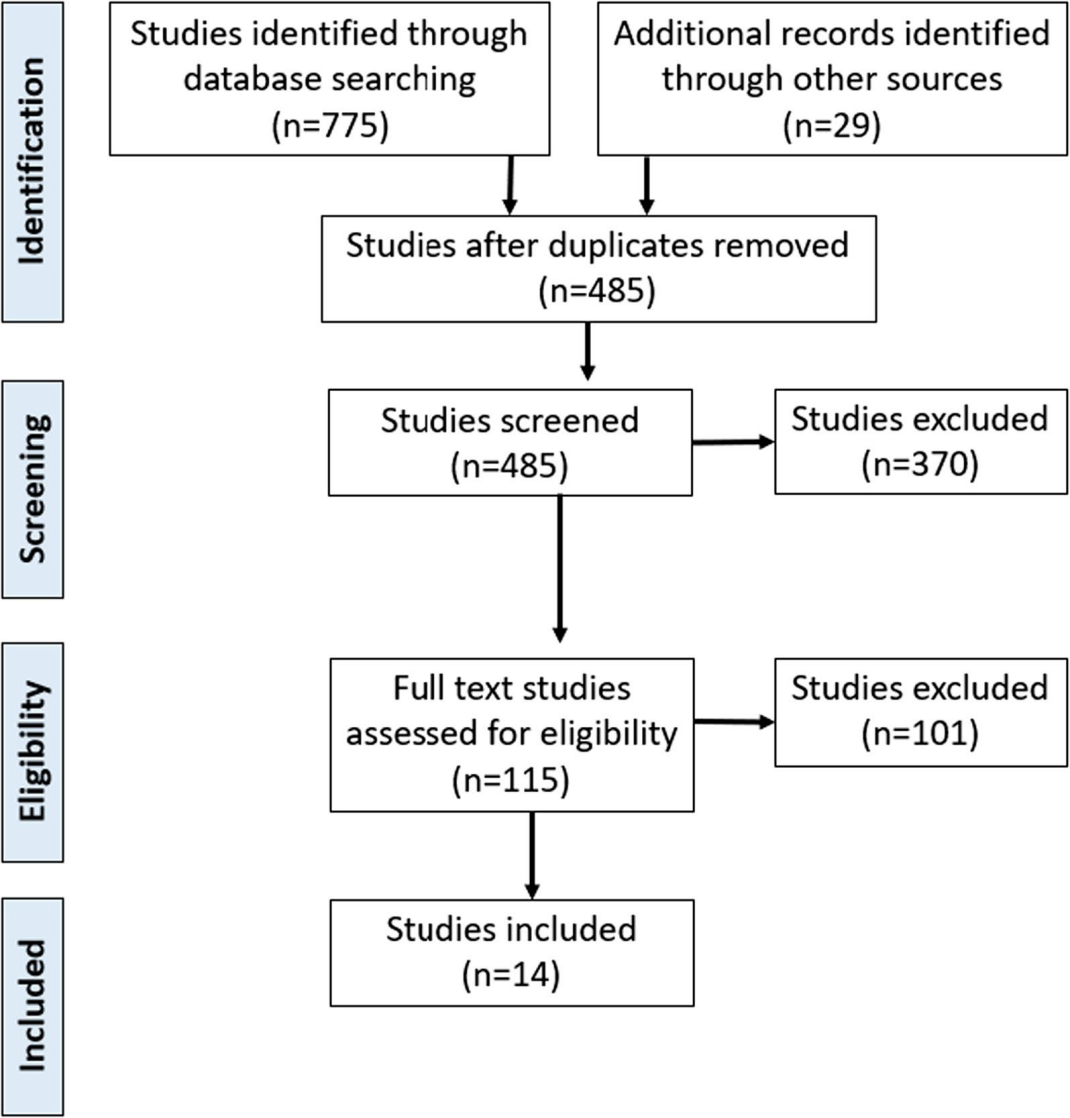
Preferred reporting items for systematic reviews and meta-analyses (PRISMA) flowchart of selection of studies

The search produced 775 studies, which were downloaded into the reference manager, Endnote. After removing duplicates, two authors (CD, HC) independently screened 456 titles and abstracts for eligibility against the inclusion criteria, and then, independently reviewed the full text of 115 studies to determine the final study selection. See Fig. 1 for the study screening process. A third author was available for cases of disagreement but was not required.

### Quality assessment

Methodological quality of the included studies was initially assessed using The National Health and Medical Research Council (NHMRC) Evidence Hierarchy (2009). Following the preliminary assessment, study quality was considered in greater detail using an amalgamation of two National Institute of Health (NIH) quality assessment tools (Dunne et al. 2021). The amalgamated tool included a checklist of 15 questions which addressed sample size, instrument validity and reliability, statistical analysis and reporting, and appropriateness of the discussion. The percentage of studies that satisfied each criterion (listed in Table 1) was calculated (possible range between 0 and 100%) as was an average quality score for each type of study design. No studies were excluded based on quality scores.

**Table 1:** Quality assessment of included studies considering NHMRC evidence level

Quality criteria	Cross-sectional studies (n = 7) (%)	Quasi-experimental studies (n = 7) (%)	All studies (n = 14) (%)
Research question/objective clearly stated	100	100	100
Population inclusion criteria clearly defined	86	86	86
Intervention clearly specified and defined	NA	71	NA
Ethical approval details reported	86	71	79
Exposure and outcome measures clearly defined	100	93	96
Exposure and outcome measures are valid and reliable	79	43	61
Sample size justification, power description, variance, or effect estimate included	29	0	14
Pre and post outcome measurements included in design	NA	93	NA
Follow-up/retention outcome measurements included in design	NA	14	NA
Outcome assessors blinded to exposure status of participants	0	0	0
Appropriate statistical techniques used	100	100	100
Participant demographics clearly reported	93	100	96
P-values or effect sizes appropriately reported	86	100	93
Appropriate conclusions aligned with aim	100	100	100
Study limitations identified	100	86	93
Average study quality score	80	71	75

Quality criteria adapted from National Institutes of Health (NIH) quality assessment tool

*NHMRC* National Health and Medical Research Counci

*NA* not applicable

Cross-sectional studies are level IV on NHMRC evidence hierarchy

Quasi-experimental studies are level III-2 on NHMRC evidence hierarchy

Risk of bias was assessed using the ROBINS-E tool (2022), which investigates the causal relationship between exposures and outcomes in observational studies. The tool examines risk of bias due to confounding, exposure measurement, participant selection, post-exposure interventions, missing data, outcome measurement, and reporting of results. All study quality assessments were conducted independently by two authors (CD, HC) and disagreements resolved through consensus. A third author was available for cases of remaining disagreement but was not required.

### Data extraction

One author (CD) extracted and collated information from each study. This included study design, summary of method, summary of intervention (quasi-experimental studies only), sample size, study quality score, specific outcome being measured, and summary of relevant results. This information was used to determine the quality of evidence for each factor and should be considered when interpreting the results.

## Results

Fourteen studies fulfilled the inclusion criteria (Petrass et al. 2021; Willcox-Pidgeon et al. 2021; Peden et al. 2017; Calverley et al. 2021; Forde et al. 2020; Franklin et al. 2015; Lawson et al. 2012; Mercado et al. 2016; Moran and Gilmore 2018; Munn et al. 2021; Olaisen et al. 2018; Peden et al. 2020; Pratt et al. 2021; Terzidis et al. 2007) and encompassed research in five countries: Australia (n = 6), United States of America (n = 5), England (n = 1), Greece (n = 1) and New Zealand (n = 1). Participants were aged between 1 and 17 years, with most aged between 5 and 14 years (see Additional file 1: Table S1, for study characteristics).

### Study quality and risk of bias

Seven studies were cross-sectional in design, and seven were quasi-experimental, ranking as level IV and III-2 on the NHMRC Evidence Hierarchy, respectively. Study quality ranged from 63 to 92%, with an average of 75%. As shown in Table 1, the average quality score for cross-sectional studies was 80% (considered good), with these studies measuring aquatic competency at one point in time (at baseline, the point-prevalence), and connecting this data to demographic information. For quasi-experimental studies, changes in aquatic competency were measured pre- post-intervention alongside participant demographic information, with an average study quality score of 71% (considered fair).

Risk of bias assessments deemed six studies to be at very high risk (Forde et al. 2020; Franklin et al. 2015; Mercado et al. 2016; Munn et al. 2021; Peden et al. 2020), and eight studies to be at high risk (Petrass et al. 2021; Willcox-Pidgeon et al. 2021; Peden et al. 2017; Calverley et al. 2021; Lawson et al. 2012; Moran and Gilmore 2018; Olaisen et al. 2018; Pratt et al. 2021; Terzidis et al. 2007) (Additional file 1: Table S2). Studies were commonly subject to bias in regard to confounding, selection of participants, and measurement of exposure and outcome.

### Measurement of aquatic competence and aquatic programs

Educational interventions differed across quasi-experimental studies regarding content, duration, and study populations. Interventions were delivered intensively for five consecutive days (Forde et al. 2020; Lawson et al. 2012; Munn et al. 2021), weekly for eight to ten weeks (Petrass et al. 2021; Calverley et al. 2021; Olaisen et al. 2018), and one intervention involved a one-day water safety event (Terzidis et al. 2007). Three studies reported on tailored interventions to children with developmental disabilities (Munn et al. 2021), children living in a regional area (Calverley et al. 2021), and Spanish-speaking Latino children (Olaisen et al. 2018).

Measurement methods and metrics for aquatic competencies were heterogeneous. For all studies, swimming and water safety skills were measured through practical testing, although different benchmark standards were applied. These standards included: the VWSC (Life Saving Victoria 2022); the (Australian) National Swimming and Water Safety Benchmark (2019); and England’s National Curriculum (Swim England 2020). Some studies referred to less formal benchmarks, including the Swim Central skills assessment checklist (Forde et al. 2020), and the iCan Swim assessment (Munn et al. 2021), and others (Lawson et al. 2012; Mercado et al. 2016; Moran and Gilmore 2018; Olaisen et al. 2018; Terzidis et al. 2007). Assessments were age-dependent and explored a combination of the following skills: safe entry and exit, submersion, flotation and sculling, swimming strokes, underwater swimming, and rescue skills (Australian Water Safety Council 2021; Petrass et al. 2021; Willcox-Pidgeon et al. 2021; Calverley et al. 2021; Forde et al. 2020; Franklin et al. 2015; Mercado et al. 2016; Munn et al. 2021; Olaisen et al. 2018; Peden et al. 2020; Pratt et al. 2021).

Water safety knowledge was measured through video interviews (Moran and Gilmore 2018), written exams (Lawson et al. 2012), surveys (Petrass et al. 2021; Calverley et al. 2021; Peden et al. 2020; Terzidis et al. 2007), practical assessment/questioning (Forde et al. 2020; Franklin et al. 2015; Peden et al. 2020), and online quizzes (Peden et al. 2017). Knowledge assessments captured topics such as identifying risks, safe aquatic behaviours, signage interpretation, cardiopulmonary resuscitation (CPR), and emergency responses. Assessors for swimming and water safety skill and knowledge ranged from researchers to swimming instructors and program deliverers. Studies measured swimming skills (Willcox-Pidgeon et al. 2021; Mercado et al. 2016; Munn et al. 2021; Olaisen et al. 2018; Pratt et al. 2021), water safety knowledge (Peden et al. 2017; Lawson et al. 2012; Moran and Gilmore 2018; Terzidis et al. 2007), or both swimming skills and water safety knowledge (Petrass et al. 2021; Calverley et al. 2021; Forde et al. 2020; Franklin et al. 2015; Peden et al. 2020).

### Demographic and background factors

This review identified 18 demographic and background factors assessed in 14 studies. Demographic and background data were collected from existing databases (Willcox-Pidgeon et al. 2021; Moran and Gilmore 2018), parent and/or child questionnaires (Petrass et al. 2021; Peden et al. 2017; Calverley et al. 2021; Franklin et al. 2015; Mercado et al. 2016; Olaisen et al. 2018; Peden et al. 2020; Pratt et al. 2021; Terzidis et al. 2007), and program enrolment forms (Forde et al. 2020; Lawson et al. 2012; Munn et al. 2021). While most demographic and/or background measures were reliably sourced, some were subject to recall and/or social desirability bias, for example, asking parents to report their child’s frequency of participation in aquatic activities or visitation to aquatic locations. There was also potential for measurement error, for example, estimating a child’s SES from the school’s postcode or the child’s residential address, rather than annual household income, and potential imprecision by relying on self-reports of medical conditions or disabilities.

In the cross-sectional studies (n = 7), aquatic competence was significantly associated with 16 factors (Table 2), which reflects a relationship between swimming skills and/or water safety knowledge and these variables at a specific point in time. Gender was the most reported factor (Willcox-Pidgeon et al. 2021; Peden et al. 2017; Franklin et al. 2015; Moran and Gilmore 2018; Pratt et al. 2021), followed by age (Willcox-Pidgeon et al. 2021; Peden et al. 2017; Franklin et al. 2015), geographic residence (Willcox-Pidgeon et al. 2021; Peden et al. 2017), income/socioeconomic status (Willcox-Pidgeon et al. 2021; Moran and Gilmore 2018), school type (Peden et al. 2017; Franklin et al. 2015), and negative prior aquatic experience (NPAE) (Peden et al. 2017; Franklin et al. 2015). One study reported on Indigeneity (Franklin et al. 2015) and another on body mass (Pratt et al. 2021); however, the results were not statistically significant. There was generally consistency in the results of reported factors, most notably age, socio-economic status, school type, and NPAE. However, there were inconsistencies in aquatic competence based on gender and geographic residence. See Table 2 for more details.

**Table 2:** Factors identified from cross-sectional studies

Factor	Outcome measured	Study	Method	Sample size	Study quality score (%)	Result summary
*Demographic factors*						
Gender	Swimming skills	Pratt et al. (2021)	Aquatic motor competence (swimming skills) assessed using the Aquatic Movement Protocol and compared with demographic data collected from participants	201	79	Female children demonstrated a higher number of swimming skills than male children
	Water safety knowledge	Moran et al. (2018)	Water safety knowledge (beach safety behaviours, beach hazards, strategies to reduce risks) collected from video interviews with students and compared with demographic data obtained from their schools	790	88	No significant differences in water safety knowledge were found when analysed based on gender
		Peden et al. (2017)	Water safety knowledge and participant demographics collected through a national online quiz	4215	75	Female children demonstrated greater water safety knowledge than male children
	Both swimming skills and water safety knowledge	Willcox-Pidgeon et al. (2021)	Swim skill and water safety knowledge data collected from national database and compared with existing records of participant demographics	43,201	83	No significant swimming skills or water safety knowledge differences were identified based on gender
	Combined aquatic competence	Franklin et al. (2015)	Aquatic competence level collected from a Swim and Survive program compared with demographic information from parent-completed enrolment form	7726	63	Female children demonstrated greater aquatic competence than male children
Age	Swimming skills	Willcox-Pidgeon et al. (2021)	Swim skill and water safety knowledge data collected from national database and compared with existing records of participant demographics	43,201	83	Older children demonstrated a higher number of swimming skills than younger children (no p-value provided)
	Water safety knowledge	Peden et al. (2017)	Water safety knowledge and participant demographics collected through a national online quiz	4215	75	Older children demonstrated greater water safety knowledge than younger children
	Combined aquatic competence	Franklin et al. (2015)	Aquatic competence level collected from a Swim and Survive program compared with demographic information from parent-completed enrolment form	7726	63	Older children demonstrated greater aquatic competence than younger children
Geographic residence	Swimming skills	Willcox-Pidgeon et al. (2021)	Swim skill and water safety knowledge data collected from national database and compared with existing records of participant demographics	43,201	83	Children from metropolitan areas demonstrated a higher number of swimming skills than children from regional areas
	Water safety knowledge	Peden et al. (2017)	Water safety knowledge and participant demographics collected through a national online quiz	4215	75	No significant differences in water safety knowledge were found when analysed by geographic residence
Income/ socio-economic status	Swimming skills	Willcox-Pidgeon et al. (2021)	Swim skill and water safety knowledge data collected from national database and compared with existing records of participant demographics	43,201	83	Children from high socio-economic areas demonstrated a higher number of swimming skills than children from low socio-economic areas
Income/ socio-economic status	Water safety knowledge	Moran et al. (2018)	Water safety knowledge (beach safety behaviours, beach hazards, strategies to reduce risks) collected from video interviews with students and compared with demographic data obtained from their schools	790	88	Children from high socio-economic areas demonstrated greater knowledge of beach safety behaviours than children from low socio-economic areas. No significant differences for the identification of beach hazards or strategies to reduce risks were found
School type	Water safety knowledge	Peden et al. (2017)	Water safety knowledge and participant demographics collected through a national online quiz	4215	75	Children from private schools demonstrated greater water safety knowledge than children from non-private schools
	Combined aquatic competence	Franklin et al. (2015)	Aquatic competence level collected from a Swim and Survive program compared with demographic information from parent-completed enrolment form	7726	63	Children from private schools demonstrated greater aquatic competence than children from non-private schools
Disability/ medical condition	Combined aquatic competence	Franklin et al. (2015)	Aquatic competence level collected from a Swim and Survive program compared with demographic information from parent-completed enrolment form	7726	63	Children without a disability/ medical condition demonstrated greater aquatic competence than children with a disability/ medical condition
Ethnicity	Water safety knowledge	Moran et al. (2018)	Water safety knowledge (beach safety behaviours, beach hazards, strategies to reduce risks) collected from video interviews with students and compared with demographic data obtained from their schools	790	88	Children from European heritage demonstrated greater knowledge of beach safety behaviours and strategies to reduce risk than children of non-European heritage. No significant differences for identifying beach hazards were found
Indigeneity	Combined aquatic competence	Franklin et al. (2015)	Aquatic competence level collected from a Swim and Survive program compared with demographic information from parent-completed enrolment form	7726	63	No significant differences in aquatic competence were identified based on indigeneity
*Background factors—aquatic experience*						
Negative prior aquatic experience	Combined aquatic competence	Peden et al. (2020)	Aquatic competence level (includes both swimming skills and water safety knowledge) collected from a Swim and Survive program compared with information from parent-completed enrolment form about child’s aquatic experience	535	79	Children without a negative prior aquatic experience demonstrated greater aquatic competence than children with a negative prior aquatic experience
		Franklin et al. (2015)	Aquatic competence level collected from a Swim and Survive program compared with demographic information from parent-completed enrolment form	7726	63	Children without a negative prior aquatic experience demonstrated greater aquatic competence than children with a negative prior aquatic experience
Frequency of participation in aquatic activity	Combined aquatic competence	Franklin et al. (2015)	Aquatic competence level collected from a Swim and Survive program compared with demographic information from parent-completed enrolment form	7726	63	Children who swam frequently (at least once a fortnight) demonstrated greater aquatic competence than children who swam infrequently (less than once a fortnight)
Private swimming lessons	Combined aquatic competence	Franklin et al. (2015)	Aquatic competence level collected from a Swim and Survive program compared with demographic information from parent-completed enrolment form	7726	63	Children who attended private swimming lessons demonstrated greater aquatic competence than children who did not attend private swimming lessons
Visitation of aquatic locations	Combined aquatic competence	Franklin et al. (2015)	Aquatic competence level collected from a Swim and Survive program compared with demographic information from parent-completed enrolment form	7726	63	Children who had visited a public pool, a beach, or a lake in the previous 12 months demonstrated greater aquatic competence than children who had not visited these locations. No significant differences in aquatic competence for visitation of rivers
Parent–child agreement about child’s comfort in deep water	Swimming skills	Mercado et al. (2016)	Assessment of child’s ability to pass/fail a swimming skills test (including propelling, breath control, and front crawl). Parent–child agreement on topics (knows how to swim, perceived good swim skills, comfort in deep water) determined through parent and child-completed surveys, and compared with pass/fail data	258	92	Children who agreed with their parents that they were comfortable in deep water demonstrated a higher number of swimming skills than children who did not
Parent–child agreement about child’s perceived swim skills	Swimming skills	Mercado et al. (2016)	Assessment of child’s ability to pass/fail a swimming skills test (including propelling, breath control, and front crawl). Parent–child agreement on topics (knows how to swim, perceived good swim skills, comfort in deep water) determined through parent and child-completed surveys, and compared with pass/fail data	255	92	Children who agreed with their parents that they had good swim skills demonstrated a higher number of swimming skills than children who did not
Pool at home	Combined aquatic competence	Franklin et al. (2015)	Aquatic competence level collected from a Swim and Survive program compared with demographic information from parent-completed enrolment form	7726	63	Children with a pool at home demonstrated greater aquatic competence than children without a pool at home
*Background factors—personal/physical characteristics*						
General motor competence	Swimming skills	Pratt et al. (2021)	Aquatic motor competence (swimming skills) assessed using the Aquatic Movement Protocol and compared with demographic data collected from participants	201	79	Children with high motor competence demonstrated a higher number of swimming skills than children with low motor competence
Stature/ height	Swimming skills	Pratt et al. (2021)	Aquatic motor competence (swimming skills) assessed using the Aquatic Movement Protocol and compared with demographic data collected from participants	201	79	Taller children demonstrated a higher number of swimming skills than short children
Body mass	Swimming skills	Pratt et al. (2021)	Aquatic motor competence (swimming skills) assessed using the Aquatic Movement Protocol and compared with demographic data collected from participants	201	79	No significant differences in swimming skills were identified when analysed by body mass

The quasi-experimental studies reported on the effectiveness of an intervention for a given demographic group and may indicate which demographic groups would benefit from tailored programs. In the quasi-experimental studies (n = 7), aquatic competence was significantly associated with four factors (Table 3). Age was the most reported factor (Calverley et al. 2021; Lawson et al. 2012; Munn et al. 2021; Olaisen et al. 2018; Terzidis et al. 2007), followed by disability/medical condition (Forde et al. 2020; Munn et al. 2021), and frequency of participation in aquatic activity (Calverley et al. 2021; Olaisen et al. 2018); however, results were inconsistent between studies for age. Gender was identified as a non-significant factor (Petrass et al. 2021; Olaisen et al. 2018).

**Table 3:** Factors identified from quasi-experimental studies

Factor	Outcome measured	Study	Intervention and method	Sample size	Study quality score (%)	Result summary
*Demographic factors*						
Gender	Swimming skills	Olaisen et al. (2018)	*Intervention*: Eight-week intervention involving a maximum of 20 age-specific swimming lessons per participant. Program targeted children with Latino ethnicity and low-income background. Program curriculum included water safety knowledge, floatation, and swimming endurance *Method*: Pre- and post-program assessment of swimming skills compared with demographic data (collected from parent-completed surveys) and program attendance data	149	73	No significant differences in the acquisition of swimming skills based on gender were found from pre- to post-program
	Both swimming skills and water safety knowledge	Petrass et al. (2021)	*Intervention*: Survival swimming program delivered in regional and metropolitan schools consisting of ten one-hour sessions. Program curriculum included water safety knowledge (local aquatic environments and hazards, survival skills) and swimming skills (floatation, gliding, entry/exits, survival strokes, and rescues) *Method*: Pre- and post-program assessment of water safety knowledge (from child-completed survey) and swimming skills (through observations) compared with demographic data (from child-completed survey)	204	73	No significant differences in the acquisition of water safety knowledge or swimming skills based on gender were found from pre- to post-program
Age	Swimming skills	Olaisen et al. (2018)	*Intervention*: Eight-week intervention involving a maximum of 20 age-specific swimming lessons per participant. Program targeted children with Latino ethnicity and low-income background. Program curriculum included water safety knowledge, floatation, and swimming endurance *Method*: Pre- and post-program assessment of swimming skills compared with demographic data (collected from parent-completed surveys) and program attendance data	149	73	Older children acquired a higher number of swimming skills than younger children from pre- to post-program
	Water safety knowledge	Calverley et al. (2021)	*Intervention*: The Bush Nippers program is a ten-day program (sessions lasting one-hour) for children who live in regional or remote areas. Program curriculum includes water safety education (regarding signage recognition, and safe aquatic behaviours) and swimming skills (floating, entry/exit, rescues, survival swimming, and first aid; aligned with the Victorian Water Safety Certificate) *Method*: Pre- and post-program assessment of swimming skills (measured practically) and water safety knowledge (measured through a survey) compared with demographic data (age, gender, aquatic experience) collected through a survey	105	73	Younger children (8–9 years) acquired water safety knowledge from pre- to post-program. whereas older children (12 years) did not
		Terzidis et al. (2007)	*Intervention*: School-based educational package including an audiovisual presentation and take-home resources about water safety *Method*: Age-specific child-completed questionnaires at pre- and one-month post-program questionnaire measured water safety knowledge and attitudes, and demographic data	1,400	70	Younger children (in kindergarten) acquired water safety knowledge from pre- to post-program, whereas older children (in elementary and high school) did not
		Lawson et al. (2012)	*Intervention*: The Danger Rangers Water Safety Program is an age-specific water safety program delivered during summer holiday camps. Program curriculum includes learning about water safety rules, the role of the lifeguard, water safety devices, and floating versus sinking. The program was delivered to children of three age-groups: kindergarten, first and second grade, and third grade *Method*: Water safety knowledge was assessed at pre- and post-program, and at three-week post-program to measure retention, compared with demographic data from parent-completed enrollment form	166	63	Children of each age group acquired water safety knowledge from pre- to post-program, whereas only older children (in third grade) demonstrated retention of knowledge at three-weeks post-program
	Combined aquatic competence	Munn et al. (2021)	*Intervention*: The iCan Swim program is a five-day program (sessions lasting 45–60 min depending on age) tailored for children with developmental disabilities. The program curriculum includes safe entry/exit, breath control, strokes, floating/gliding, and rolling *Method*: Pre- and post-program assessment of swimming skills compared with medical diagnosis of child reported by parents on program enrollment forms	86	77	No significant differences in acquisition of aquatic competence based on age were found from pre- to post-program
Geographic residence	Both swimming skills and water safety knowledge	Petrass et al. (2021)	*Intervention*: Survival swimming program delivered in regional and metropolitan schools consisting of ten one-hour sessions. Program curriculum included water safety knowledge (local aquatic environments and hazards, survival skills) and swimming skills (floatation, gliding, entry/exits, survival strokes, and rescues) *Method*: Pre- and post-program assessment of water safety knowledge (from child-completed survey) and swimming skills (through observations) compared with demographic data (from child-completed survey)	204	73	Children from metropolitan areas acquired both water safety knowledge and swimming skills from pre- to post-program. Children from regional areas acquired swimming skills from pre- to post-program but did not acquire water safety knowledge
Disability/ medical condition	Swimming skills	Munn et al. (2021)	*Intervention*: The iCan Swim program is a five-day program (sessions lasting 45–60 min depending on age) tailored for children with developmental disabilities. The program curriculum includes safe entry/exit, breath control, strokes, floating/gliding, and rolling *Method*: Pre- and post-program assessment of swimming skills compared with medical diagnosis of child reported by parents on program enrollment forms	86	77	Children with ASD and ADHD acquired a higher number of swimming skills from pre- to post-program than children with only ASD
	Combined aquatic competence	Forde et al. (2020)	*Intervention*: The SWIM Central program is a ten-day program (sessions lasting 30 min). Program curriculum includes water safety knowledge (Never Swim Alone/Call for Help/Reach, Throw, Don’t Go!) and swimming skills (safe entry, safe exit, jump in and kick to wall, freestyle arm stroke, and floating on back) *Method*: Pre- and post-program assessment of water safety knowledge and swimming skills, compared with demographic data (child’s age, ethnicity, diagnosis) from parent-completed enrollment form	76	63	Children with disabilities showed significant increases in one skill (floating on back) from pre- to post-program. Children without disabilities showed significant increases in six skills from pre- to post-program
*Background factors—Aquatic experience*						
Frequency of participation in aquatic activity	Swimming skills	Olaisen et al. (2018)	*Intervention*: Eight-week intervention involving a maximum of 20 age-specific swimming lessons per participant. Program targeted children with Latino ethnicity and low-income background. Program curriculum included water safety knowledge, floatation, and swimming endurance *Method*: Pre- and post-program assessment of swimming skills compared with demographic data (collected from parent-completed surveys) and program attendance data	149	73	Children who swam frequently (attended ≥ 10 lessons) acquired a higher number of swimming skills than children who swam less frequently (attended ≤ 7 lessons) from pre- to post-program
	Water safety knowledge	Calverley et al. (2021)	*Intervention*: The Bush Nippers program is a ten-day program (sessions lasting one-hour) for children who live in regional or remote areas. Program curriculum includes water safety education (regarding signage recognition, and safe aquatic behaviours) and swimming skills (floating, entry/exit, rescues, survival swimming, and first aid; aligned with the Victorian Water Safety Certificate) *Method*: Pre- and post-program assessment of swimming skills (measured practically) and water safety knowledge (measured through a survey) compared with demographic data (age, gender, aquatic experience) collected through a survey	105	73	Children who swam frequently (at least twice a month) acquired greater water safety knowledge than children who swam less frequently (less than twice a month) from pre- to post-program

## Discussion

Given the benefits and popularity of swimming, (World Health Organisation 2014) it is of concern that many primary school aged children are not meeting standard aquatic competency benchmarks (Willcox-Pidgeon et al. 2021). Accordingly, it is important to understand the demographic and background factors that may be associated with the demonstration and acquisition of aquatic competence. Findings from the current systematic review add to the body of knowledge through identifying 16 demographic and background factors significantly associated with the development of children’s aquatic competence. Relatively stronger evidence was evident for age, socio-economic status, school type, NPAE, disability and medical condition, and frequency of participation in aquatic activity. The significance of these factors was identified based on large participant numbers in at least two studies. Evidence for the remaining factors was weaker and should be further explored in future research. Further work is also required to tailor the list of factors to each HIC as swimming and water safety skill development opportunities vary between HIC nations.

In cross-sectional studies, there was robust evidence supporting the finding that children from lower socioeconomic families and from non-private schools displayed lower aquatic competence. Whilst not explicitly identified as a confounding variable, socioeconomic status, geographic residence, and education levels could be associated with accessibility to swimming facilities and engagement in swimming lessons. This notion is supported through recent reviews which reported disparities in physical activity participation for both children and adults based on education levels and socioeconomic status (Lõhmus et al. 2022; Juneau et al. 2015; Pearson et al. 2022). A challenge for the aquatic sector, as well as governments and non-profit organisations, is to better understand the interplay between socio-economic status and geographic residence with accessibility to, and engagement in swimming. Understanding this relationship is critical, as consistent with previous research (Bullough et al. 2015; Chan et al. 2020; Santibañez-Gutierrez et al. 2022), factors such as participating in private swimming lessons, having a pool at home, frequency of aquatic participation, and recent visitation to aquatic locations were all identified in this review as positively associated with the development of aquatic competence. Although living in a regional area was associated with children demonstrating lower practical skills when compared to those living in metropolitan areas, no significant difference was found in the level of water safety knowledge. It should be noted that demographic and background factors are likely to influence the acquisition of practical swimming skills and water safety knowledge in different ways, and these outcomes should be considered independently in future research.

Children who experience barriers to practicing and participating in aquatics, for example financial or physical, are likely to display lower aquatic competency. This finding has important implications for the aquatic industry, particularly as swimming competency is not considered a static personal ability (Swimming 2014). It is an acquisition process, which is built on practice and experience in aquatic environments (Rocha et al. 2018). Thus, providing strategies and opportunities for children to engage in regular swimming, and in a variety of aquatic environments, is likely to enhance aquatic competency. Several studies recommended solutions to address lower aquatic competence among children living in a regional area, including: the provision of educational technology to teach applied water safety lessons (Peden et al. 2017); adapting coastal programs for inland residents (Calverley et al. 2021); co-designing programs with communities; and working closely with ‘community champions’ to encourage participation and uptake of programs (Beattie et al. 2008). Future research should continue to examine and evaluate the effects of such programs on acquisition of aquatic competence, to provide more pertinent evidence regarding effectiveness of tailored programs and to identify which demographic and background factors respond best to this approach. In addition, less formal enablers of swimming frequency have been identified, including having a friend that enjoys swimming; being encouraged to swim by parents; and having parents who could swim well (Willcox-Pidgeon et al. 2020). It is also important to better understand whether these personal and social factors are related to the development and acquisition of aquatic competence.

Regarding child-specific demographic and background factors, current findings showed poorer aquatic competency among younger children, which is consistent with findings from previous studies (Irwin et al. 2009; Moran et al. 2012). Taller children showed significantly greater aquatic competency compared to shorter children, although in most cases there would be a positive correlation between child age and height which may explain why height was a significant factor. Disagreement between quasi-experimental studies that explored the association between age and water safety knowledge may be attributed to heterogeneous interventions and the age brackets considered for the studies. The effect of gender, where girls demonstrated significantly higher aquatic competency in respect to knowledge and skills compared to boys, contrasts with previous findings (Chan et al. 2020; Santibañez-Gutierrez et al. 2022). This may be due to the inclusion criteria for this review, which required a practical measure of aquatic competency. It has been reported that when self-estimating aquatic competence, males may overestimate their ability and underestimate their risk of drowning (Petrass et al. 2012). Future research should continue to explore gender differences to understand how this factor is associated with the development of children’s aquatic competence, particularly considering intersectionality with other factors such as cultural background, age, and socio-economic status. Where possible, quasi-experimental studies should be conducted to explore the acquisition and retention of aquatic competency for children of different genders and ages.

It is well established that individuals with pre-existing medical conditions or disabilities are at an elevated drowning risk (World Health Organisation 2014), particularly those with autism spectrum disorder and epilepsy (Peden and Willcox-Pidgeon 2020). Peden and Franklin (2020) further identified that having a pre-existing medical condition significantly increased the likelihood of reporting a NPAE. Much of the previous disability-related literature has focused on children who are high-functioning and neglected to explore the development of effective programs for children who are non-verbal, or those with multiple conditions who may be uniquely marginalised from participating in lessons (Pearn and Franklin 2013; Kraft and Leblanc 2018). It should be acknowledged that children with disabilities/medical conditions are not a homogenous group, and that it is difficult to interpret the findings of this review given limited information on the nature and severity of participant diagnoses. Nevertheless, the quasi-experimental studies in this review identified that with appropriate tailoring, children with disabilities can develop their aquatic competence (Forde et al. 2020; Munn et al. 2021), yet additional work is required to implement differentiation to ensure swimming lessons are inclusive and accessible and support skill attainment for all children. For example, Martin and Dillenburger (2019) demonstrated that for children with autism spectrum disorder, sessions could be differentiated regarding the skills taught to students, teaching methods employed and duration and frequency of lessons, although recognised that differentiation needed to be tailored to the individual (Martin and Dillenburger 2019). Whilst research has indicated that the type of negative experience a child has encountered may dictate the severity of fear of drowning and impact their willingness to learn and engage in programs (Peden et al. 2020; Mische Lawson et al. 2019), there were insufficient studies that met the inclusion criteria to explore the impact of specific NPAE on the development of aquatic competence. Accordingly, further prospective research should explore how specific NPAEs impact the development of aquatic competence.

Learning to swim is a recognised drowning prevention strategy, and this review has highlighted several factors that are significantly associated with the development of aquatic competence, which may place individuals at risk of not achieving age-expected benchmarks. Thus, focusing prevention efforts on individuals at increased risk may be timely. Whilst we are not suggesting that all groups require tailored programs, the findings of this study should be used by swim schools and learn-to-swim teachers to ensure the practice of differentiation is applied to ensure lessons are personalised for groups of children with varying levels of ability, interest, and readiness (Gibbs and McKay 2021). Whipp et al. (2014) have offered practical ways to incorporate differentiated instruction into children’s swimming lessons, which include differentiation of content, processes, and product. Some include the segregation of a class based on ability and the selected delivery of appropriate activities to each group, the use of specialised equipment, delivering lessons in alternative environments and conditions for children to perform skills in, and the use of non-traditional methods, such as ongoing observation, for students to demonstrate their learning (Whipp et al. 2014). In addition to work from swim schools, parents and caregivers need to be educated and aware of the importance of developing sound aquatic competence, and strategies are required to ensure the message reaches identified at-risk groups from this review. Finally, at all levels of government, at-risk groups should be prioritised to receive swimming and water safety education, and education departments should assist in supporting the development and/or delivery of evidence-based, tailored programs to these groups.

While this study advances our understanding and has identified groups which may require greater focus in future drowning prevention efforts, findings must be considered in the light of some limitations. First, the inclusion of articles published in English, and from HIC may explain the limited identification of factors regarding culture and language. It is well established that culturally and linguistically diverse people are at heightened drowning risk (), and thus exploring the impact of ethnicity and language barriers on aquatic competence is pertinent. Further research is required to determine the transferability of risk factors to LMIC. Secondly, whilst we reported on methodological quality and risk of bias, study quality of included studies was moderate. To address this concern, future prospective research should provide detailed information regarding the methodological approach to offer context about the swimming program and data collection measures and assessment procedures for aquatic competency. Where possible, researchers should seek to implement valid and reliable measures of demography and aquatic competency. Third, all identified factors were listed independently as part of the results, although relationships could exist between some variables (e.g. age and height; socio-economic status and attendance at private school). Development of a greater evidence base in this area will enable statistical analysis to ascertain where collinearity may exist between factors. Efforts to improve the quality, rigour, and comparability of future studies will strengthen the evidence base around the association between demographic and background factors and children’s aquatic competence.

## Conclusions

This review has identified several factors that are significantly associated with the development of children’s aquatic competence, in particular, age, socio-economic status, school type, NPAE, disability and medical condition, and frequency of participation in aquatic activity. These findings reinforce the importance of differentiated instruction within aquatic programs to cater to the needs and strengths of individual students. This review should assist in informing the development, delivery, and evaluation of swimming and water safety programs that aim to develop aquatic competencies.

### Supplementary Information

**Additional file 1**: **Table S1** Study characteristics. **Table S2** Risk of bias assessment results.

## Data Availability

Not applicable.

## Abbreviations

WHO: World Health Organization
SES: Socio-economic status
PRISMA: Preferred Reporting Items for Systematic Reviews and Meta-Analyses
VWSC: Victorian Water Safety Certificate
HIC: High-income countries
LMIC: Low- and middle-income countries
NHMRC: National Health and Medical Research Council
NIH: National Institutes of Health
CPR: Cardio-pulmonary resuscitation
NPAE: Negative prior aquatic experience
ASD: Autism Spectrum Disorder
ADHD: Attention Deficit Hyperactivity Disorder
