# Supplementary material for: A systematic review of demographic and background factors associated with the development of children’s aquatic competence
Source: Inj Epidemiol. 2023 Aug 8;10:42. doi: 10.1186/s40621-023-00447-4 (PMC10408087; doi:10.1186/s40621-023-00447-4)
Supplement: Supplementary file 1 — Additional file 1: Table S1 Study characteristics. Table S2 Risk of bias assessment results. [file 40621_2023_447_MOESM1_ESM.docx]

**Table S1** Study characteristics

| **Study** | **Study design**  **(NHMRC evidence hierarchy)** | **Sample size** | **Study quality score (%)** | **Country** | **Participants and program** | **Demographic or background data** | **Aquatic competence data** | **Statistical analysis** |
| --- | --- | --- | --- | --- | --- | --- | --- | --- |
| Mercado MC, Quan L, Bennett E, Gilchrist J, Levy BA, Robinson CL, et al. Can you really swim? Validation of self and parental reports of swim skill with an in-water swim test among children attending community pools in Washington State. Injury prevention: journal of the International Society for Child and Adolescent Injury Prevention. 2016;22(4):253-60. | Cross-sectional  (IV) | 258 | 92 | United States of America | Children aged 7-17 years who attended selected public pools on data collection days. | Eight trained interviewers delivered verbal surveys to both parent and child. Surveys included four child swim skill measures: knowing how to swim (yes/ no), participated in swimming lessons (yes/ no), perceived good swim skills (good/ not good), and comfort in deep water (comfortable/ uncomfortable). | Children underwent a swim test upon entering the pool. Skills assessed were propulsion, breath control, and freestyle stroke. Children either passed or failed this test. | Pearson’s r used to determine which paired child-parent reports of swim skill measures were correlated with the child passing the swim test. |
| Moran K, Gilmore A. Children’s Understanding of Water Safety and Perceptions of Risk at the Beach. New Zealand Journal of Educational Studies. 2018;53(2):227-39. | Cross-sectional  (IV) | 790 | 88 | New Zealand | Children aged 8-9 years from schools that participated in a health and physical education study. Eight children randomly samples from each school to participate. | Demographic data (gender, ethnicity, socio-economic status) obtained from their schools. | Children undertook Water Safety task, a series of activities and questions during a one-on-one video. Questions assessed water safety knowledge (beach safety behaviours, beach hazards, strategies to reduce risks). | Participant responses (representing their water safety knowledge) analysed against their demographics using Chi square tests. |
| Willcox-Pidgeon SM, Peden AE, Scarr J. Exploring children's participation in commercial swimming lessons through the social determinants of health. Health Promotion Journal of Australia. 2021;32(2):172-81. | Cross-sectional  (IV) | 43,201 | 83 | Australia | Children aged 5-12 who had participated in commercial swimming lessons in three Australian states. | Demographic data (gender, socio-economic status, and geographic residence) extracted from existing participant records. | Swimming skills assessed according to the Australian National Swimming and Water Safety Education Framework. Skills included entry and exit; sculling and body orientation, movement and swimming strokes; survival and personal flotation skills; underwater skills; rescue skills; | Chi-squared analysis between demographic data and swimming skills. |
| Pratt NA, Duncan MJ, Morris MG, Oxford SW. The Reliability and Validation of the Aquatic Movement Protocol as an Instrument for Assessing Aquatic Motor Competence in Primary Aged Children. Journal of Motor Learning and Development. 2021;9(3):512-25. | Cross-sectional  (IV) | 201 | 79 | England | Children aged 7-9 years from primary schools invited to participate in research; testing the validity of an Aquatic Movement Protocol. | Anthropometric data (height and body mass) collected and general motor competence assessed using the Test of Gross Motor Development-2. Participants were categorised as having low, medium, or high general motor competence. | Swimming skills including swimming strokes, gliding, sculling, treading water, submersion, and entry/exit were assessed, according to Swim England’s national curriculum. A researcher performed skill assessments. | Pearson product moment correlations used to analyse general and aquatic motor competence. |
| Peden AE, Franklin RC. Learning to swim: An exploration of negative prior aquatic experiences among children. International Journal of Environmental Research and Public Health. 2020;17(10). | Cross-sectional  (IV) | 535 | 79 | Australia | Children aged 5-12 years enrolled in a Swim and Survive program in Canberra, Australia. The program is a 10-lesson program delivered by the Royal Life Saving Society as part of public primary school education. | Parent-completed enrolment form collected information on whether their child had had a negative prior aquatic experience. Researchers coded this data into 11 categories of experiences. | Swimming skills and water safety knowledge were assessed (against Water Safety Education Competency Framework). Each participant was assigned a ‘level,’ which denoted their combined aquatic competence. Skills include safe entry/exit, sculling, swimming and survival strokes, floating, diving, rescues, and knowledge of aquatic dangers. | Analysis of variance was used to determine the influence of negative prior aquatic experiences on swim level achieved. |
| Munn EE, Ruby L, Pangelinan MM. Improvements in swim skills in children with autism spectrum disorder following a 5-day adapted learn-to-swim program (iCan swim). Journal of Clinical Medicine. 2021;10(23). | Quasi-experimental  (III-2) | 86 | 77 | United States of America | Children aged 3-16 years with a disability (ASD, ADHD, cerebral palsy, epilepsy, etc). participating in the iCan Swim program. This is a five-day program (sessions lasting 45-60 minutes depending on age) tailored to children with developmental disabilities. | Parents reported their child’s medical diagnosis as part of the enrollment process into the program. | Pre- and post-program assessment of swimming skills. Skills assessed included safe entry/exit, breath control, strokes, floating/gliding, and rolling. | Follow-up T tests were used to determine differences in swim skills between children of different diagnoses. |
| Peden AE, Franklin RC; and Scarr, J. Measuring Australian Children’s Water Safety Knowledge: The National Water Safety Quiz. International Journal of Aquatic Research and Education: 2017;10(4). | Cross-sectional  (IV) | 4,215 | 75 | Australia | Children aged 5-12 years who had completed an online water safety quiz during school hours. | The quiz collected demographic data from participants, including gender, age, school type, and postcode. | Water safety knowledge was assessed through the quiz, which consisted of 41 questions, about signage, CPR, different aquatic environments, rescues, and survival strokes. Participants received a score for the quiz, denoting the percentage of questions they answered correctly. | Chi-square and analysis of variance were used to determine differences in water safety knowledge between demographic groups. |
| Calverley HLM, Birch R, Strugnell G, Santiago AC, Baker S, Matthews BL. Bush Nippers: Evaluating the effectiveness of the Nipper education program in regional inland Victoria, Australia. Health Promotion Journal Australia. 2021. | Quasi-experimental  (III-2) | 105 | 73 | Australia | Children aged 8-12 years who participated in Bush Nippers. This is a ten-day program (sessions lasting one-hour) for children who live in regional or remote areas. | A survey administered to children collected demographic data, including their age, gender, and frequency of participation in aquatic activity). | Pre- and post-program assessments of water safety knowledge (through a survey) and swimming skills (through practical assessments); aligned with the Victorian Water Safety Certificate. | Paired samples t-tests identified differences in knowledge between pre- and post-program for each demographic group. |
| Olaisen RH, Flocke S, Love T. Learning to swim: role of gender, age and practice in Latino children, ages 3-14. Injury prevention: journal of the International Society for Child and Adolescent Injury Prevention. 2018;24(2):129-34. | Quasi-experimental  (III-2) | 149 | 73 | United States of America | Children aged 3-14 years participating in a program targeted at children with Latino ethnicity and low-income background. Program is an eight-week intervention involving a maximum of 20 age-specific swimming lessons per participant. | Parent-completed surveys collected demographic data, including age, gender, and ethnicity of the child. The number of lessons each child attended was also recorded. | Swimming skills were assessed at pre- and post-program, skills assessed included floating, endurance, and safety skills. | Chi-squared test and analysis of variance to determine differences in swimming skills based on demographics and attendance. |
| Petrass LA, Simpson K, Blitvich J, Birch R, Matthews B. Exploring the impact of a student-centred survival swimming programme for primary school students in Australia: the perceptions of parents, children and teachers. European Physical Education Review. 2021;27(3):684-702. | Quasi-experimental  (III-2) | 204 | 73 | Australia | Children aged 10-14 across one regional and one metropolitan school, participating in a Swim and Survive Program in Victoria. Program consisted of ten one-hour sessions. | Demographic information (geographic residence, age, gender, perceived aquatic skills) was collected in a child-completed survey at pre-program. | Practical assessments included water safety knowledge (local aquatic environments and hazards, survival skills through child-completed survey) and swimming skills (floatation, gliding, entry/exits, survival strokes, and rescues, through practical assessments). These assessments occurred at pre- and post-program. | Mann–Whitney U tests used to determine differences between pre- and post-program skills/ knowledge between groups. |
| Terzidis A, Koutroumpa A, Skalkidis I, Matzavakis I, Malliori M, Frangakis CE, et al. Water safety: age-specific changes in knowledge and attitudes following a school-based intervention. Injury Prevention (1353-8047). 2007;13(2):120-4. | Quasi-experimental  (III-2) | 1,400 | 70 | Greece | Children aged 5-15 years from schools in Athens invited to undertake water safety testing. This followed a school-based educational package including an audiovisual presentation and take-home resources about water safety. | Demographic information (age, gender, number of siblings, material education, knowledge of swimming) collected in age-specific child-completed questionnaire. | Age-specific child-completed questionnaires at pre- and one-month post-program measured water safety knowledge and attitudes. | General linear models and the Wald test for heterogeneity used to test for differences in skills among different age groups. |
| Forde AL, Zeman EA, Clarke L. Effectiveness of an intensive drowning prevention program and skills retention by children with and without disabilities. International Journal of Aquatic Research and Education. 2020;12(2). | Quasi-experimental  (III-2) | 76 | 63 | United States of America | Children aged 3-11 years, who attended the SWIM Central program, a ten-day program (sessions lasting 30 minutes). | Demographics (age, ethnicity, diagnosis) collected from a parent-completed enrollment form. | Skills assessed include water safety knowledge (Never Swim Alone/Call for Help/Reach, Throw, Don’t Go!) and swimming skills (safe entry/exit, freestyle arm stroke, and floating on back) | Wilcoxon signed rank test used to compare pre- and post-program scores for both groups. |
| Franklin RC, Peden AE, Hodges S, Lloyd N, Larsen P, O'Connor C, et al. Learning to swim: What influences success? International Journal of Aquatic Research and Education. 2015;9(3):220-40. | Cross-sectional  (IV) | 7,726 | 63 | Australia | Children aged 5-12 years enrolled in a Swim and Survive program in Canberra, Australia. The program is a 10-lesson program delivered by the Royal Life Saving Society as part of public primary school education. | Parent-completed enrolment form collected information on child’s age, sex, school grade, disability, Indigeneity, aquatic experience, and perceived swimming ability. | Swimming skills and water safety knowledge were assessed (against Water Safety Education Competency Framework). Each participant was assigned a ‘level,’ which denoted their combined aquatic competence. Skills include safe entry/exit, sculling, swimming and survival strokes, floating, diving, rescues, and knowledge of aquatic dangers. | Pearson’s chi-square and analysis of variance between demographic/ background factor and combined aquatic competence. |
| Lawson KA, Duzinski SV, Wheeler T, Yuma-Guerrero PJ, Johnson KM, Maxson RT, et al. Teaching safety at a summer camp: evaluation of a water safety curriculum in an urban community setting. Health Promotion Pract. 2012;13(6):835-41. | Quasi-experimental  (III-2) | 166 | 63 | United States of America | Children aged 3-9 years who participated in the Danger Rangers Water Safety Program; an age-specific water safety program delivered during summer holiday camps | Parent-completed enrollment forms collected demographic data (age, aquatic experience, ethnicity). | Water safety knowledge assessed at pre-, post- and three weeks post-program through written exams. Topics explored included water safety rules, the role of the lifeguard, water safety devices, and floating versus sinking. | Paired t-tests were used to compare mean overall exam scores and demographics data**.** |

*Note.* Studies ranked in descending study quality score order

NHMRC = National Health and Medical Research Council

**Table S2** Risk of bias assessment results

|  | Risk of bias due to confounding | Risk of bias arising from measurement of the exposure | Risk of bias in selection of participants | Risk of bias due to post-exposure interventions | Risk of bias due to missing data | Risk of bias arising from measurement of the outcome | Risk of bias in selection of reported result | Overall risk of bias |
| --- | --- | --- | --- | --- | --- | --- | --- | --- |
| Franklin et al., 2015 | High | Concerns | Concerns | Low | High | High | Low | Very high |
| Moran et al., 2018 | High | Very high | Concerns | Low | Low | Concerns | Low | Very high |
| Peden et al., 2020 | High | Very high | Concerns | Low | Concerns | Low | Low | Very high |
| Peden et al., 2017 | High | Low | Concerns | Low | Low | Low | Low | High |
| Willcox-Pidgeon et al., 2021 | High | Concerns | Concerns | Low | Concerns | Concerns | Low | High |
| Pratt et al., 2021 | High | Low | Concerns | Low | Low | Concerns | Low | High |
| Calverley et al., 2021 | High | Concerns | Concerns | Low | Concerns | Concerns | Low | High |
| Forde et al., 2020 | High | Low | Very high | Low | Low | High | Low | Very high |
| Lawson et al., 2012 | High | Low | Concerns | Low | Low | High | Concerns | High |
| Mercado et al., 2012 | High | Low | Low | Low | High | Low | Low | Very high |
| Munn et al., 2021 | High | Low | High | Low | Very high | Concerns | Low | Very high |
| Olaisen et al., 2018 | High | Concerns | Low | Low | Concerns | Concerns | Low | High |
| Petrass et al., 2021 | Low | Low | Concerns | Low | Concerns | High | Low | High |
| Terzidis et al., 2007 | High | Low | Concerns | Low | Low | High | Low | High |

*Note*. Categories for risk of bias judgements: Low risk (Low), Some concerns (Concerns), High risk (High), Very high risk (Very high)
